# The role of grammatical role and thematic role predictability in reference form production in Mandarin Chinese

**DOI:** 10.3389/fpsyg.2022.930572

**Published:** 2022-08-04

**Authors:** Heeju Hwang, Suet Ying Lam, Wenjing Ni, He Ren

**Affiliations:** Department of Linguistics, The University of Hong Kong, Pokfulam, Hong Kong SAR, China

**Keywords:** null pronouns (zero anaphors), overt pronouns, reference production, grammatical role, thematic role, predictability, Chinese

## Abstract

Evidence suggests that English speakers use pronouns when referring to the grammatical subject and predictable thematic role. We tested how grammatical role and thematic role predictability affect different types of referential forms, namely, overt pronouns and null pronouns in Mandarin Chinese. We found that both overt and null pronouns were sensitive to grammatical role. However, we did not find any evidence that overt and null pronouns were sensitive to thematic role predictability. Although null pronouns were influenced by grammatical role, the rate of null pronouns for subject reference was very low compared to that of overt pronouns. Given the frequent occurrence of null pronouns in Mandarin, our results suggest that the use of null pronouns may not be explained by a simple grammatical role mechanism.

## Introduction

It is generally agreed that entities in a discourse differ in salience. At a particular point in the discourse, some entities are more salient or accessible than other entities in the speaker’s mental model of the discourse. Much of the research on reference production suggests that referential form choice is driven by the salience or accessibility of an entity; a more reduced referential form (e.g., pronoun) tends to be used for a highly accessible entity, while a more explicit referential form (e.g., name) tends to be used for a less accessible entity (e.g., Chafe, 1976; Givón, 1983; Ariel, 1990; Gundel et al., 1993). The factors that have been claimed to influence the salience of an entity (and thereby the likelihood of that entity being subsequently referred to with a reduced form) include structural (e.g., grammatical role), semantic (e.g., thematic role predictability), and discourse-level factors (e.g., topicality) (e.g., Givón, 1983; Arnold, 2001; Rohde and Kehler, 2014; Rosa and Arnold, 2017; see Arnold, 2010 for a review of the factors that increase the salience of the referent). The goal of the current study is to test how different types of referential forms are influenced by structural (i.e., grammatical role) and semantic factors (i.e., thematic role predictability).

Many theories of reference production suggest that structural factors such as grammatical role play a key role in the choice of reference form [henceforth structural account, e.g., Ariel’s (1990) Accessibility Theory, Grosz et al. (1995) Centering Theory; Kehler et al. (2008) Bayesian model]. In English, it is well established that speakers tend to use pronouns when the referent is in subject position of the preceding clause than in non-subject position (i.e., the subjecthood effect, e.g., Frederiksen, 1981; Fletcher, 1984; Brennan et al., 1987; Gordon et al., 1993; Stevenson et al., 1994; Brennan, 1995; Nakatani, 1997; Arnold et al., 2000; Arnold, 2001; Fukumura and van Gompel, 2010; Rohde and Kehler, 2014). For example, in (1) a pronoun is the most natural way to refer to the subject character (Ana in 1a, Liz in 1b), while a name is more acceptable for reference to the non-subject character (Liz in 1a, Ana in 1b).


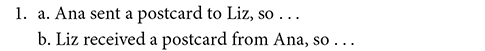


Evidence suggests that grammatical role also plays a key role in referential form choice in agreement pro-drop languages with rich agreement morphology such as Italian, Spanish, and Greek. For example, Miltsakaki (2007) found that Greek speakers used more null pronouns to refer to subject referents and more overt pronouns to refer to non-subject referents in main clause continuations (see Carminati, 2002; de Carvalho Maia et al., 2017 for Italian; Alonso-Ovalle et al., 2002; Filiaci et al., 2014 for Spanish for similar findings in comprehension).

The grammatical role effect could stem from information structure and/or syntax. It is commonly suggested that subjecthood is correlated with topicality. Although the grammatical subject does not necessarily serve the role of sentence or discourse topic (e.g., the sentence *No one amazes Liz* is about *Liz* rather than *no one*), topical entities tend to be realized in subject position (e.g., Kieras, 1981; Reinhart, 1981; Chafe, 1994; Lambrecht, 1994). If the salience of grammatical role is derived from topicality, the grammatical role effect could be attributed to information structural differences between subjects and non-subjects, not to the grammatical roles *per se* (e.g., Ariel, 1990; Gundel et al., 1993; Lambrecht, 1994; Grosz et al., 1995).

However, the grammatical role effect could be also rooted in syntax (e.g., Kieras, 1979; Crawley et al., 1990). It is well established that subject assignment is closely linked to conceptual or lexical accessibility of an entity (see Bock, 1982; McDonald et al., 1993; Ferreira and Slevc, 2007; Jaeger and Norcliffe, 2009; for reviews of factors that assign subject assignment). Accessible entities are retrieved sooner during the sentence production process and are positioned in earlier and higher grammatical positions. For example, animate entities are highly accessible and tend to be assigned to the subject function (e.g., Clark, 1965; Harris, 1978; Bock and Warren, 1985; see Branigan et al., 2008 for a review). Similarly, when a patient entity (e.g., cow) in a transitive event (e.g., a horse kicking a cow) was made accessible by a semantic prime (e.g., milk, e.g., Bock, 1986) or a subliminal visual cue that directed speakers’ initial attention to the patient entity (e.g., Gleitman et al., 2007; Hwang and Kaiser, 2015), speakers were more likely to produce a passive sentence that mentioned the patient entity in subject position (e.g., *a cow was kicked by a horse*). If a more accessible entity tends to be realized in subject position, the grammatical role effect could be linked to conceptual or lexical accessibility of the grammatical subject.

In addition to structural factors, some theories of reference production suggest that referential form choice is also influenced by semantic information such as referent predictability (expectations about what entities will be mentioned next) (henceforth predictability account). For example, Arnold’s (1998, 2001) Expectancy Hypothesis suggests that speakers use reduced forms such as pronouns for entities that are predictable (see also Givón, 1983, 1988, 1989). When completing sentence fragments, speakers are likely to mention entities in certain thematic roles again. For example, in (1a) and (1b) which depict transfer events, speakers are more likely to continue with the goal (Liz) than the source (Ana). This is because the goal as the endpoint of transfer events is likely to be a natural starting point for what happens next (e.g., Stevenson et al., 1994; Arnold, 2001; Rohde and Kehler, 2014; Simpson et al., 2016). Similarly, in (2a) and (2b) in which the stimulus (Ana) is the assumed cause of the events (i.e., implicit causality), speakers are more likely to continue with the stimulus (Ana) than the experiencer (Liz) (e.g., Brown and Fish, 1983; Au, 1986; Stevenson et al., 1994; Crinean and Garnham, 2006; Hartshorne et al., 2015).


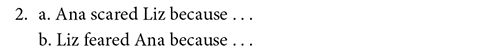


These biases toward certain thematic roles are closely tied to inter-sentential connectives or the coherence relations between the sentences (e.g., Ehrlich, 1980; Au, 1986; Stevenson et al., 1994, 2000). The expectations of the goal reference in (1) and the stimulus reference in (2) are generated by the connectives *so* and *because*, respectively. If the sentences in (1) continue with *because*, the preference for the goal is reduced. In (2), the experiencer is preferred when the sentences continue with *so*.

Critically, Arnold’s (1998, 2001) Expectancy Hypothesis suggests that entities that are likely to be mentioned again are accessible and tend to be referred to with pronouns (see also Givón, 1983, 1988). For example, Rosa and Arnold (2017) found that English speakers used more pronouns when referring to the goal than the source following sentences like (1) (see also Arnold, 2001; Weatherford and Arnold, 2021). Kaiser et al. (2011), however, provide evidence against the Expectancy Hypothesis. They found that given passive prompts such as *Mary was hit by Kate at school. As a result*…, English speakers were equally likely refer to the agent (Kate) and the patient (Mary). Yet they used pronouns more to refer to the patient than the agent. Kaiser et al. (2011) suggest that thematic role affects pronoun use, but predictability is not directly linked to the likelihood of pronominalization.

If thematic role predictability does affect reference form production, it exerts a relatively weak influence compared to grammatical role. Rosa and Arnold (2017) found that the predictability effect did not overturn the subjecthood effect and was detected only when participants showed variation in their referential form choice. Furthermore, predictability did not influence referential form choice for implicit causality (IC) verbs in numerous studies (Kehler et al., 2008; Fukumura and van Gompel, 2010; Rohde and Kehler, 2014; but see Weatherford and Arnold, 2021). For example, Fukumura and van Gompel (2010) found that English speakers were more likely to refer to the stimulus (Ana) when asked to complete sentence fragments such as (2). However, they did not use more pronouns for the stimulus than the experiencer. Based on the results, Fukumura and van Gompel suggest that structural factors such as grammatical role is the sole determinant of reference form [see also Kehler et al.’s (2008) Bayesian model, Kehler and Rohde, 2013].

In sum, the findings in English suggest that both grammatical role and thematic role predictability contribute to referential form choice, but grammatical role plays a central role in the use of pronouns; the predictability effect may work on top of the grammatical role effect and may be limited to certain contexts.

Yet it is not clear how well the structural account and the predictability account that are primarily based on pronouns in English extend to other forms of reference and to other languages. The present study addresses this issue by investigating how grammatical role and thematic role predictability influence the use of overt and null pronouns in Mandarin Chinese.

Prior work on narratives suggests that referential form choice in a discourse pro-drop language is sensitive to the grammatical role of the referent. For example, Clancy and Downing (1987) found that Japanese speakers tended to use null pronouns when referring to subject referents in spoken narratives. More recently, Ngo (2019) showed that Vietnamese speakers primarily used null pronouns for subject referents in both spoken and written narratives. Experimental research provides further evidence for the effect of grammatical role. Hwang (2018, 2020) conducted a series of sentence completion studies in Cantonese and Mandarin and found that overt pronouns were more common for subject referents than non-subject referents. Consistent with the structural account, the results of these studies suggest that grammatical role plays an important role in referential form choice in a discourse pro-drop language. Yet they do not provide an adequate basis for assessing the grammatical role effect because they did not control for predictability. Using a sentence completion task, Hwang (in press) recently found that Korean speakers used more null pronouns for subject referents than non-subject referents while controlling for predictability (see also Hwang, 2022 for a similar finding). However, the study did not test the effect of grammatical role on overt pronouns, and thus leaves open the question of how grammatical role affects different types of referential form available in a given language.

Compared to the grammatical role effect, much less is known about how thematic role predictability affects the use of overt and null pronouns in a discourse pro-drop language. In a recent study, Zhan et al. (2020) found that thematic role predictability did not influence Mandarin speakers’ decision to use a pronoun. Mandarin speakers were more likely to use pronouns for subject referents, but not for predictable entities. The results, however, do not provide conclusive evidence about the effect of predictability because the authors only examined IC verbs, for which many previous studies did not find any predictability effect. They also did not distinguish between overt pronouns and null pronouns in their analyses. Thus, it is not clear whether the effect of predictability differs between the two types of pronouns.

In sum, it is not clear how grammatical role and thematic role predictability influence referential form choice in a discourse pro-drop language. The present study examines the role of grammatical role and thematic role predictability in the production of overt and null pronouns in Mandarin. Mandarin provides a good testing ground for evaluating the validity of the structural account and the predictability account against both overt and null pronouns. This is because unlike in some discourse pro-drop languages such as Korean and Japanese, in which overt pronouns are rare (e.g., Kim, 1989 for Korean; Clancy, 1980, 1982 for Japanese), both overt and null pronouns are frequently used in Mandarin (e.g., Chen, 1986; Christensen, 2000).

To test the effects of grammatical role and thematic role predictability on referential form choice in Mandarin, we manipulated the grammatical role and the predictability of a thematic role using transfer verbs (e.g., give/receive) and IC verbs (e.g., impress/admire). Each transfer and IC verb item was followed by *suoyi* “so” in one condition and *yinwei* “because” in the other condition. We used these verbs because they allow us not only to see whether the predictability effect differs depending on the verb type, but also to distinguish the grammatical role effect from the predictability effect and vice versa (Rosa and Arnold, 2017). For example, when IC verbs are followed by the connective *because*, continuations are expected to describe the cause of the event, and the stimulus is more predictable than the experiencer. Importantly, some IC verbs (e.g., impress, scare) place the predictable stimulus referent in subject position (N1-biased, e.g., *Ana impressed Liz*), while others (e.g., admire, fear) place the stimulus referent in non-subject position (N2-biased, e.g., *Liz admired Ana*). Thus, the grammatical role effect can be assessed while controlling for thematic role predictability, and the predictability effect can be assessed while controlling for grammatical role.

Following previous studies on the effects of grammatical role and thematic role predictability on referential form choice (e.g., Stevenson et al., 1994; Arnold, 2001; Fukumura and van Gompel, 2010; Rohde and Kehler, 2014), we employed a written sentence completion task. The participants’ task was to provide a plausible continuation to each sentence fragment.

If overt and null pronouns are sensitive to grammatical role as suggested by the structural account, we predict that Mandarin speakers would use overt and null pronouns more for the subject of the preceding clause than the non-subject. This should lead to a main effect of grammatical role on overt and null pronouns.

If overt and null pronouns are also sensitive to thematic role predictability as suggested by the predictability account, we predict that Mandarin speakers would use overt and null pronouns more for the predictable thematic role than the less predictable thematic role. For transfer verbs, we predict that the goal referent would be more predictable following “so,” and thus would be more likely to be pronominalized than the source referent following “so.” However, we expect that the goal preference should be reduced following “because” and the rate of pronominalization toward the goal should decrease accordingly. For IC verbs, we expect that the stimulus role would be predictable following “because” whereas the experiencer role would be predictable following “so.” This predicts that the stimulus referent would be more likely to be pronominalized following “because,” whereas the experience referent would be more likely to be pronominalized following “so.” This should result in an interaction between thematic role and connective for both transfer and IC verbs.

## Methods

### Participants

Sixty-three native Mandarin speakers voluntarily participated in the experiment. They were recruited over the internet. One participant was excluded for providing nonsensical continuations. This left 62 participants in the analysis (43 females; *M*_*age*_ = 23.4, Range = 18–30).

### Materials and procedure

We constructed 48 experimental items. 24 items were designed with transfer verbs, and 24 with IC verbs. Transfer items described a transfer event involving two human characters in the roles of goal and source. The goal role was in subject position for half the items (3a) and in non-subject position for the other half (3b).


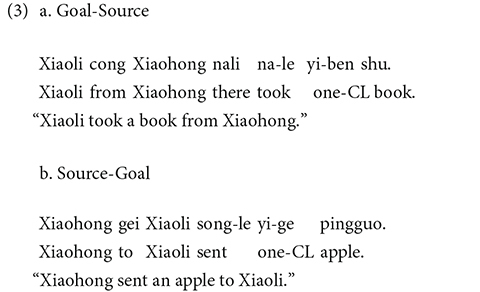


For IC verbs items involving stimulus and experiencer roles, the stimulus role was in subject position for half the items (4a) and in non-subject position for the other half (4b).


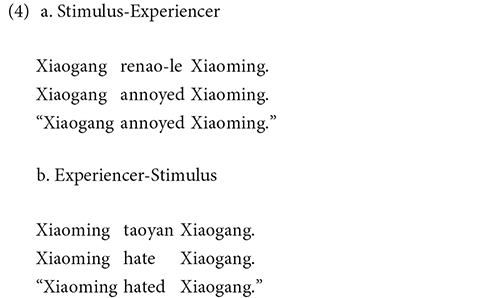


Transfer and IC verb items consisted of the two participants of the same gender, counterbalanced between male (Xiaogang and Xiaoming) and female characters (Xiaohong and Xiaoli). Two female and two male characters occurred in subject and non-subject position an equal number of times. Each of transfer and IC verb items was followed by *suoyi* “so” in one condition and *yinwei* “because” in the other condition.

In addition to the 48 target items, we constructed 48 filler items using verbs other than transfer verbs and IC verbs (e.g., intransitive verbs). The fillers described an event involving a single character or two characters using a similar structure as the experimental items [i.e., X did something (with Y)].

We created two lists using a Latin Square design, in which each participant was exposed to each item in only one condition but encountered all conditions across different items. Participants were randomly assigned to one of the two lists.

The study was administered as an online survey using *Qualtrics*. Participants were instructed to provide a plausible continuation to each sentence fragment with one of the characters in the story. Before proceeding to the main experiment, participants were presented with two example trials and four practice trials.

### Scoring

Chinese is a topic-prominent language, in which the topic-comment relation plays a major role (e.g., Li and Thompson, 1976). Unlike a subject-prominent language such as English, Chinese commonly allows a topic-comment construction (5a), in which a sentence topic (e.g., that piece of land) precedes the grammatical subject (e.g., rice). We coded the first element of each response, which was either the sentence topic in a topic-comment structure or the grammatical subject in a subject-predicate structure (see also Hwang, in press; Lam and Hwang, accepted).


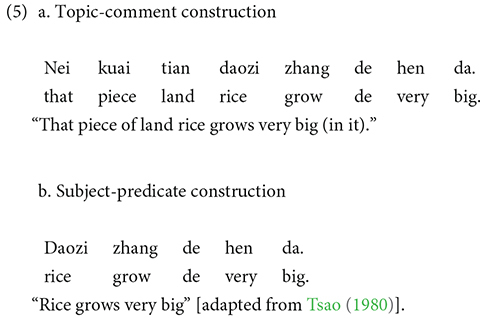


Responses were coded for (a) choice of referring expressions (null pronoun, overt pronoun, or name), (b) grammatical role of referents in the preceding sentence (subject vs. non-subject) and (c) thematic role of referents (goal/source for transfer verbs and stimulus/experiencer for IC verbs).

Utterances were excluded from the analysis if (a) participants referred to more than one character at once (e.g., *ta-men yiqi huijia-le* “they came home together”); (b) they referred to entities other than the prompt characters (e.g., *Xiaoming de mama shengbing-le* “Xiaoming’s mother was sick”); (c) they produced an erroneous response in which the referent did not match the meaning of the continuation (e.g., *Xiaogang xiaohua Xiaoming. Suoyi Xiaogang hen shengqi*. “Xiaogang teased Xiaoming. So Xiaogang was angry.”); or (d) they produced an ambiguous utterance in which the intended referent could not be determined by semantic context (e.g., *Xiaoli gei Xiaohong juan-le yixie yiwu. Yinwei ta zhang-pang-le.* “Xiaoli donated some clothes to Xiaohong. This was because she got fat.”). About 8% of trials (476 out of 5,828 trials) were removed for one of the above reasons, resulting in 5,352 trials in the analysis.

### Analysis

We performed separate analyses for transfer and IC verb items. For each verb type, we first analyzed whether the choice of referent (subject vs. non-subject) was affected by verb bias (N1-biased vs. N2-biased) and connective (“because” vs. “so”) as reported in the literature. The results were analyzed using logit mixed effects models (Baayen et al., 2008; Jaeger, 2008). The analyses were conducted with the lme4 R package (Bates et al., 2015). We fitted maximal random effects structure, including main effects and the interaction. If the fully maximal model did not converge, we simplified models until convergence was achieved (Barr et al., 2013). We report the coefficient for each independent variable and its level of significance for the final model. Coefficients are given in log-odds.

We then analyzed whether referential form choice (names vs. overt pronouns vs. null pronouns) was affected by grammatical role (subject vs. non-subject) and thematic role predictability (interaction between thematic role and connective). The three-way choice of referential form was analyzed using a mixed-effects categorical logistic regression model. We implemented a Bayesian model using brms R package (Bürkner, 2017). We chose a weakly informative prior, using the Cauchy distribution with center 0 and scale 2.5, as recommended by Gelman et al. (2008). The Bayesian regression model provided a posterior distribution of the outcome. We report the estimated mean, the estimated error, and the 95% Credible Interval (CrI) of this posterior distribution in log odds. The 95% CrI represents a 95% of probability that the outcome lies in the boundary of this interval (van de Schoot et al., 2014). If the interval does not contain zero and the limits of the interval are all positive or negative, it is considered to be equivalent to a significant effect in frequentist statistics.

## Results

### Choice of referent

**FIGURE 1:**
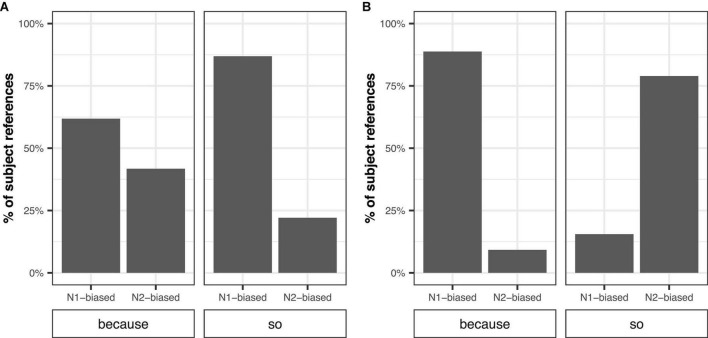
Percentages of subject reference by verb type [subject (N1)-biased vs. object (N2)- biased] and connective (“because” vs. “so”) for transfer verbs **(A)** and IC verbs **(B)**.

Figure 1 plots percentages of subject reference by verb type and connective. When transfer verbs were followed by the connective “so,” Mandarin speakers referred to subject (goal) referents more following N1-biased verbs (86.9%) and non-subject (goal) referents more following N2-biased verbs (77.9%). This indicates that Mandarin speakers referred to the goal more than the source for both N1 and N2-biased transfer verbs following “so.” When transfer verbs were followed by the connective “because,” however, the preference for the goal was reduced. The rate of subject (goal) reference was 61.8% for N1-biased transfer verbs and the rate of non-subject (goal) reference was 58.2% for N2-biased transfer verbs.

When IC verbs were followed by “because,” Mandarin speakers referred to subject (stimulus) referents more following N1-biased IC verbs (88.8%) and non-subject (stimulus) referents more following N2-biased IC verbs (90.8%). The preference for the stimulus was reversed when IC verbs were followed by “so.” Mandarin speakers referred to non-subject (experiencer) referents more following N1-biased IC verbs (84.5%) and subject (experiencer) referents more following N2-biased IC verbs (78.9%). This indicates that Mandarin speakers tended to refer to the stimulus following “because,” but the experiencer following “so.” These patterns of reference for transfer and IC verbs were consistent with the results in the literature (e.g., Stevenson et al., 1994; Fukumura and van Gompel, 2010; Simpson et al., 2016).

We analyzed response frequencies of referents (Subject = 1, Non-subject = 0) as a function of verb bias (sum-coded: Subject-biased = 0.5, Object-biased = −0.5) and connective (sum-coded: Because = −0.5, So = 0.5 for transfer verbs and Because = 0.5, So = −0.5 for IC verbs). For both transfer and IC verb responses, the maximal model to converge included verb bias, connective and their interaction as fixed effects, random intercepts for participants and items and a random slope for connective by items.

We found a significant interaction between verb bias and connective for both transfer (Table 1.1) and IC verb responses (Table 1.2), confirming that the effect of verb bias was modulated by connective. We also found a significant effect of verb bias for both verb types, such that Mandarin speakers referred to subjects more following N1-biased verbs than N2-biased verbs.

**TABLE 1:** Summary of logit mixed effect models for referent choice.

Fixed effects	Coefficient	SE	Wald Z	*p*
**1. Transfer verb responses**				
Intercept	0.26	0.19	1.34	0.18
Verb bias	2.56	0.35	7.25	<0.001
Connective	0.34	0.31	1.10	0.27
Verb bias × Connective	3.15	0.63	4.99	<0.001
**2. IC verb responses**				
Intercept	−0.18	0.25	−0.73	0.46
Verb bias	0.56	0.39	1.96	0.05
Connective	0.17	0.33	0.52	0.60
Verb bias × Connective	10.20	0.75	13.51	<0.001

### Choice of referential form

#### Transfer verbs

**FIGURE 2:**
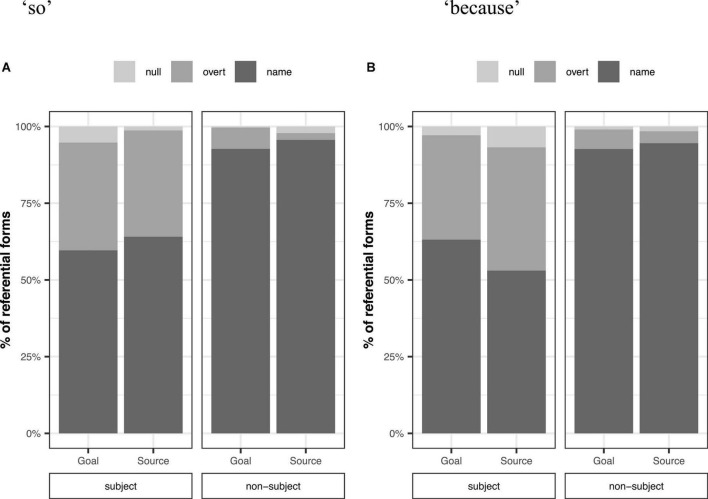
Percentages of referential forms by grammatical role (subject vs. non-subject) and thematic role following “so” **(A)** and “because” **(B)** for transfer verbs.

Figure 2 plots percentages of referential forms for subjects and non-subjects as a function of thematic role following “so” (a) and “because” (b) for transfer verbs. In terms of grammatical role, Mandarin speakers used both overt and null pronouns more when referring to subjects than non-subjects (Overt pronouns: 35.7 vs. 5.8%, Null pronouns: 4.5 vs. 0.9%).

Mandarin speakers, however, did not use reduced expressions more when referring to the predictable thematic role. Although the goal was more predictable than the source following “so” and “because,” participants tended to use both overt and null pronouns more for the source than the goal (Overt pronouns: 23.0 vs. 21.1%, Null pronouns: 3.5 vs. 2.5%).

To examine how referential form choice was affected by grammatical role and thematic role predictability, we analyzed referential forms (names vs. overt pronouns vs. null pronouns) as a function of grammatical role (sum-coded: Subject = 0.5, Non-subject = −0.5), thematic role (sum-coded: Goal = 0.5, Source = −0.5), connective (sum-coded: Because = 0.5, So = −0.5), and the interaction between thematic role and connective. We hypothesized that the effect of thematic role would be modulated by connective, and thus included the interaction between thematic role and connective in the model. However, the interaction between grammatical role and thematic role, the interaction between grammatical role and connective, and the three-way interaction between grammatical role, thematic role, and connective were not our main theoretical interests. Thus, to avoid overfitting, these interactions were not included in the model.

We chose names as the reference level. We implemented a full model which included grammatical role, thematic role, connective, the interaction between thematic role and connective as fixed predictors, random intercepts for participants and items, and random slopes of grammatical role, thematic role, connective, the interaction between thematic role and connective for both participants and items. The model was fitted using 3 chains, each with iterations of 4,000 of which the first 600 are warmup to calibrate the sampler, resulting in 10,200 posterior examples.

We found a main effect of grammatical role on overt and null pronouns compared to names (Table 2.1). Mandarin speakers used more overt and null pronouns for subjects than non-subjects. There was no effect of thematic role or connective on overt and null pronouns. For overt pronouns, the interaction between thematic role and connective was significant. We analyzed the effects of grammatical role and thematic role separately for “*so*” and “*because*.” However, we did not find any effect of thematic role for either connective. The interaction between thematic role and connective was not significant for null pronouns. These results suggest that Mandarin speakers did not use reduced forms to refer to predictable referents.

**TABLE 2:** Summary of the Bayesian mixed-effects categorical logistic regression model for the choice of referential form (95% credible intervals that do not contain zero, i.e., equivalent to significance in frequentist statistics, are bolded).

Predictor	Estimated mean	Estimated error	95% CrI
**1. Transfer verbs**			
**1.1 Overt pronouns vs. Names**			
Intercept	–4.16	0.67	**[**−**5.59,**−**2.97]**
Grammatical role	4.93	0.95	**[3.34, 7.07]**
Thematic role	0.50	0.47	[−0.43, 1.43]
Connective	0.16	0.35	[−0.52, 0.85]
Thematic role × Connective	–1.41	0.70	**[**−**2.81,**−**0.09]**
**1.2 Null pronouns vs. Names**			
Intercept	–11.38	2.47	**[**−**17.27,**−**7.62]**
Grammatical role	3.72	1.93	**[0.09, 7.80]**
Thematic role	–0.81	1.24	[−3.40, 1.55]
Connective	–0.06	1.10	[−2.36, 2.03]
Thematic role × Connective	–1.40	1.87	[−5.46, 2.03]
**2. Implicit causality verbs**			
**2.1 Overt pronouns vs. Names**			
Intercept	–3.70	0.63	**[**−**5.05,**−**2.55]**
Grammatical role	4.17	0.70	**[2.98, 5.74]**
Thematic role	–0.50	0.53	[−1.59, 0.51]
Connective	–0.39	0.52	[−1.44, 0.65]
Thematic role × Connective	0.99	1.01	[−0.88, 3.11]
**2.2 Null pronouns vs. Names**			
Intercept	–15.86	4.11	**[**−**25.91,**−**9.91]**
Grammatical role	6.34	2.91	**[1.45, 12.66]**
Thematic role	–1.14	1.71	[−4.80, 2.05]
Connective	–2.09	1.67	[−5.78, 0.79]
Thematic role × Connective	–1.72	2.65	[−7.53, 2.98]

#### Implicit causality verbs

**FIGURE 3:**
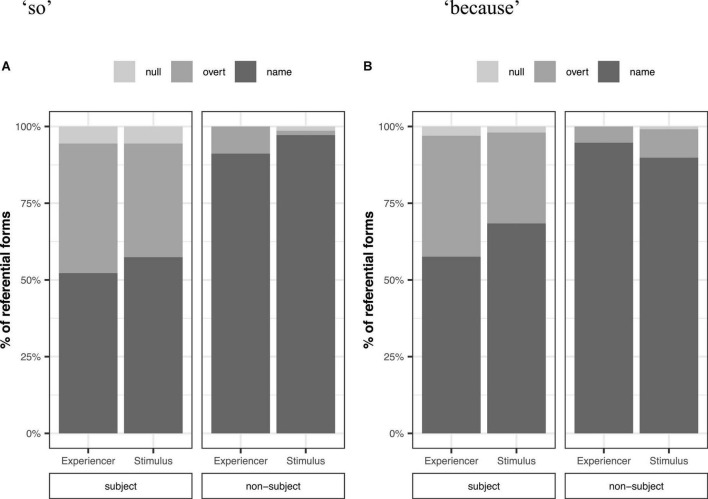
Percentages of referential forms by grammatical role (subject vs. non-subject) and thematic role following “so” **(A)** and “because” **(B)** for IC verbs.

Figure 3 shows percentages of referential forms for subjects and non-subjects as a function of thematic role following “so” (a) and “because” (b) for IC verbs. Similar to transfer verbs, Mandarin speakers used more overt and null pronouns for subjects than non-subjects (Overt pronouns: 35.9 vs. 8.1%, Null pronouns: 3.7 vs. 0.5%).

In contrast to grammatical role, thematic role predictability did not yield consistent results. Although the stimulus was more predictable than the experiencer following “because,” overt pronouns were more common for the experiencer (21.1%) than the stimulus (19.0%). The rate of null pronouns was similar between the experiencer (1.4%) and the stimulus (1.4%). Following “so,” the experiencer was more predictable than the stimulus. The rate of overt pronouns was higher for the experiencer (24.8%) than the stimulus (16.7%), but the rate of null pronouns was not higher for the experiencer (2.7%) than the stimulus (3.2%).

To examine how referential form choice was affected by grammatical role and thematic role predictability, we analyzed referential forms (names vs. overt pronouns vs. null pronouns) as a function of grammatical role (sum-coded: Subject = 0.5, Non-subject = −0.5), thematic role (sum-coded: Stimulus = 0.5, Experiencer = −0.5 in IC verbs), connective (sum-coded: Because = 0.5, So = −0.5), and the interaction between thematic role and connective. The results of the analysis revealed a main effect of grammatical role on overt and null pronouns (Table 2.2). However, we did not find any effect of thematic role, connective, or the interaction between the two.

In sum, the results of the study suggest that grammatical role had a significant effect on both overt and null pronouns. However, we did not find any evidence that thematic role predictability played a role in the use of overt and null pronouns.

## Discussion

The present study set out to evaluate the validity of the structural account and the predictability account in the use of overt and null pronouns in a discourse pro-drop language, namely Mandarin Chinese. We found that both overt and null pronouns were sensitive to grammatical role. However, we did not find any evidence that thematic role predictability affected the two types of pronouns.

The results of the study suggest that grammatical role plays an important role in determining referential form consistent with the structural account [e.g., Ariel’s (1990) Accessibility Theory, Grosz et al.’s (1995) and Fukumura and van Gompel (2010) Centering Theory; Kehler et al.’s (2008) Bayesian model]. Yet the results suggest that grammatical role alone is not sufficient to account for different types of referential forms. Although grammatical role had a significant effect on the use of null pronouns, the rate of null pronouns for subjects was very low compared to that of overt pronouns in the study. Given that null pronouns occur frequently in Mandarin, our findings indiate that grammatical role is not likely to be a single mechanism that underlies the use of null pronouns. That is, there are likely other constraints than grammatical role that must be met to license the use of null pronouns.

This raises the question of what are the factors that drive speakers to choose a null pronoun over other forms in a discourse pro-drop language. The results of our recent research suggest that null pronouns are sensitive to discourse-level factors such as topicality (Lam and Hwang, accepted; see also Hwang, in press for the role of discourse connection or continuity). For example, Lam and Hwang (accepted) found that Mandarin speakers used overt and null pronouns more when referring to subjects than non-subjects. Critically, they found that Mandarin speakers used more null pronouns but fewer overt pronouns when referring to more topical subjects. These findings suggest that subjecthood cannot be subsumed under topichood, and that subjecthood and topichood play a distinct role in referential form production in Mandarin. If the use of null pronouns is driven by both structural and discourse-level factors, null pronouns may not frequently occur in contexts where the discourse factors are not clearly present or manipulated. More work is needed to determine the precise nature and factors that drive overt and null pronouns, as well as the underlying mechanisms.

In contrast to grammatical role, thematic role predictability did not affect Mandarin speakers’ choice of referential form. This finding is consistent with the literature that suggests that thematic role predictability is unrelated to referential form production (e.g., Kehler et al., 2008; Fukumura and van Gompel, 2010; Kaiser et al., 2011; Rohde and Kehler, 2014; Lam and Hwang, accepted), but incompatible with the prior work that suggests that thematic role predictability affects pronoun use (e.g., Arnold, 2001; Rosa and Arnold, 2017; Weatherford and Arnold, 2021). Note, however, that the predictability effect, if attested, was relatively weak compared to the grammatical role effect. The predictability effect did not consistently emerge for subjects and non-subjects and was likely to emerge only when participants showed variation in their referring expressions. The subtle effect of predictability could be harder to detect when speakers have more flexibility in their choice of referential form as in Mandarin (overt pronouns, null pronouns, and names) compared to English (pronouns and names). Overall, it seems likely that thematic role predictability effects are limited to certain contexts or language, and referential form production cannot be explained by a predictability mechanism. Future work could illuminate the nature of the predictability effect by better characterizing the conditions under which thematic role predictability affects referential form production.

Our results, taken together with the results of comprehension research in the literature, may suggest that reference production and comprehension are not mirror images of each other (e.g., Kehler et al., 2008; Rohde and Kehler, 2014). Previous research on comprehension using transfer and IC verbs suggests that overt and null pronouns are equally subject-biased (e.g., Kim et al., 2013) or null pronouns have a stronger subject bias in discourse pro-drop languages (e.g., Ueno and Kehler, 2016). Our finding that participants used overt pronouns more than null pronouns for subject referents seems to contradict the findings in the comprehension literature. This, however, can be easily explained if we look at the proportions of subject reference of overt and null pronouns, i.e., how often overt and null pronouns each refer to subjects compared to non-subjects. The proportions of subject reference were slightly higher for null pronouns (85.3%) than overt pronouns (83.8%) in our study. If null pronouns refer to subjects as often as or more often than overt pronouns, comprehenders may interpret null pronouns as referring to subjects as often as or more often than overt pronouns, accounting for the subject bias of overt and null pronouns reported in comprehension research. This suggests that comprehension patterns of overt and null pronouns may not predict their relative frequencies in production and vice versa, indicating an asymmetry between reference production and comprehension.

In sum, our investigation of referential form choice in Mandarin showed that both overt and null pronouns were sensitive to grammatical role in support of the structural account. Yet the low rate of null pronouns for subject reference suggests that a single grammatical role mechanism is not likely to drive different types of referential forms. In contrast to grammatical role effect, we did not find any evidence that thematic role predictability affected referential form production. This further suggests that referential form production cannot be explained by a predictability mechanism.

## Data availability statement

The raw data supporting the conclusions of this article will be made available by the authors, without undue reservation.

## Ethics statement

The studies involving human participants were reviewed and approved by the Human Research Ethics Committee, The University of Hong Kong. The patients/participants provided their written informed consent to participate in this study.

## Author contributions

HH developed the study concept. HH, SL, WN, and HR designed the study. SL, WN, and HR collected the data. HH and SL analyzed and interpreted the data and drafted the manuscript. All authors approved the final version of the manuscript for submission.
