# Supplementary material for: The role of grammatical role and thematic role predictability in reference form production in Mandarin Chinese
Source: Front Psychol. 2022 Aug 4;13:930572. doi: 10.3389/fpsyg.2022.930572 (PMC9386568; doi:10.3389/fpsyg.2022.930572)
Supplement: Supplementary file 1 [file Data_Sheet_1.docx]

**Appendix**

Experimental materials

1.Transfer verb items

1.1. Source-goal items

1. 小刚给小明汇了一笔钱。因为/所以……

Xiaogang remitted some money to Xiaoming. This is because/So…

1. 小明给小刚寄了一包腊肠。因为/所以……

Xiaoming sent a bag of sausage to Xiaogang. This is because/So…

1. 小丽给小红提供了那批货。因为/所以……

Xiaoli offered the cargo to Xiaohong. This is because/So…

1. 小红向小丽丢了一个钱袋。因为/所以……

Xiaohong threw a wallet to Xiaoli. This is because/So…

1. 小红给小丽送了一个苹果。因为/所以……

Xiaohong gave an apple to Xiaoli. This is because/So…

1. 小丽给小红捐了一些衣物。因为/所以……

Xiaoli donated some clothes to Xiaohong. This is because/So…

1. 小刚给小明留下了一笔钱。因为/所以……

Xiaogang left a sum of money to Xiaoming. This is because/So…

1. 小红向小丽卖了一台电脑。因为/所以……

Xiaohong sold a computer to Xiaoli. This is because/So…

1. 小刚向小明还了一个篮球。因为/所以……

Xiaogang returned a basketball to Xiaoming. This is because/So…

1. 小明给小刚递了一包方便面。因为/所以……

Xiaoming passed a pack of instant noodles to Xiaogang. This is because/So…

1. 小明给小刚带了一个蛋糕。因为/所以……

Xiaoming brought a cake to Xiaogang. This is because/So…

1. 小丽给小红送了一瓶水。因为/所以……

Xiaoli gave Xiaohong a bottle of water. This is because/So…

1.2. Goal-source items

1. 小红从小丽那里收到了一盒巧克力。因为/所以……

Xiaohong received a box of chocolate from Xiaoli. This is because/So…

1. 小明从小刚那里拿回了这支笔。因为/所以……

Xiaoming got a pen back from Xiaogang. This is because/So…

1. 小刚从小明那里收到了一些书。因为/所以……

Xiaogang received some books from Xiaoming. This is because/So…

1. 小刚从小明那里拿了一双筷子。因为/所以……

Xiaogang took a pair of chopsticks from Xiaoming. This is because/So…

1. 小明从小刚那里继承了一套房子。因为/所以……

Xiaoming inherited a house from Xiaogang. This is because/So…

1. 小丽从小红那里拿了一本书。因为/所以……

Xiaoli got a book from Xiaohong. This is because/So…

1. 小明从小刚那里收到了一笔款项。因为/所以……

Xiaoming received an amount of money from Xiaogang. This is because/So…

1. 小红从小丽那里收到了一些物资。因为/所以……

Xiaohong received some supplies from Xiaoli. This is because/So…

1. 小红从小丽那里拿到了一箱水果。因为/所以……

Xiaohong got a box of fruits from Xiaoli. This is because/So…

1. 小刚从小明那里拿了一盒饭。因为/所以……

Xiaogang took a plate of rice from Xiaoming. This is because/So…

1. 小丽从小红那里买了一套房子。因为/所以……

Xiaoli bought a house from Xiaohong. This is because/So…

1. 小丽从小红那里接了一个皮球。因为/所以……

Xiaoli caught a ball from Xiaohong. This is because/So…

2. Implicit causality verb items

2.1. Experiencer-stimulus items

1. 小刚笑话小明。因为/所以……

Xiaogang teased Xiaoming. This is because/So…

1. 小明敬佩小刚。因为/所以……

Xiaoming admired Xiaogang. This is because/So…

1. 小红害怕小丽。因为/所以……

Xiaohong feared Xiaoli. This is because/So…

1. 小刚欣赏小明。因为/所以……

Xiaogang appreciated Xiaoming. This is because/So…

1. 小明讨厌小刚。因为/所以……

Xiaoming disliked Xiaogang. This is because/So…

1. 小丽鄙视小红。因为/所以……

Xiaoli despised Xiaohong. This is because/So…

1. 小丽喜欢小红。因为/所以……

Xiaoli liked Xiaohong. This is because/So…

1. 小红憎恨小丽。因为/所以……

Xiaohong hated Xiaoli. This is because/So…

1. 小明嫌弃小刚。因为/所以……

Xiaoming disliked Xiaogang. This is because/So…

1. 小刚崇拜小明。因为/所以……

Xiaogang adored Xiaoming. This is because/So…

1. 小红厌恶小丽。因为/所以……

Xiaohong detested Xiaoli. This is because/So…

1. 小丽注意到了小红。因为/所以……

Xiaoli noticed Xiaohong. This is because/So…

2.2. Stimulus-experiencer items

1. 小丽吓到了小红。因为/所以……

Xiaoli frightened Xiaohong. This is because/So…

1. 小红诱惑了小丽。因为/所以……

Xiaohong tempted Xiaoli. This is because/So…

1. 小红吸引小丽。因为/所以……

Xiaohong attracted Xiaoli. This is because/So…

1. 小明惊艳了小刚。因为/所以……

Xiaoming amazed Xiaogang. This is because/So…

1. 小刚烦到了小明。因为/所以……

Xiaogang bothered Xiaoming. This is because/So…

1. 小丽冒犯了小红。因为/所以……

Xiaoli offended Xiaohong. This is because/So…

1. 小刚启发了小明。因为/所以……

Xiaogang inspired Xiaoming. This is because/So…

1. 小丽激怒了小红。因为/所以……

Xiaoli irritated Xiaohong. This is because/So…

1. 小明逗乐了小刚。因为/所以……

Xiaoming amused Xiaogang. This is because/So…

1. 小刚惹恼了小明。因为/所以……

Xiaogang annoyed Xiaoming. This is because/So…

1. 小明感动了小刚。因为/所以……

Xiaoming moved Xiaogang. This is because/So…

1. 小红恶心到了小丽。因为/所以……

Xiaohong disgusted Xiaoli. This is because/So…
